# Supplementary material for: Non-Selective Evolution of Growing Populations
Source: PLoS One. 2015 Aug 14;10(8):e0134300. doi: 10.1371/journal.pone.0134300 (PMC4537121; doi:10.1371/journal.pone.0134300)
Supplement: S1 Text — Using the theory of Pólya urns, we calculate exactly the steady state values of: (i) the distribution of population compositions x, (ii) its mean value, and (iii) its variance. (PDF) [file pone.0134300.s002.pdf]

S1 Text

Calculation of probability distribution. Each population in the ensemble is initialized with A_0 individuals of type A and B_0 of type B. In the general case, A_0 and B_0 are independent random variables for each population with distributions $P(A_0)$ and $P(B_0)$. All populations evolve for ΔN reproduction events, of which a random amount ΔA generate new A-individuals. From the mathematical literature [35], it is well-known that ΔA follows a beta-binomial, with A_0, B_0 and ΔN as parameters. The fraction of A-individuals x, then follows the probability

$$P(x) = \sum_{A_0,B_0} P(A_0)P(B_0)P(\Delta A = x(A_0 + B_0 + \Delta N) - A_0|A_0, B_0, \Delta N)\,, \quad (3)$$

where the sums run over all allowed values of their respective indices.
$P(\Delta A = k|A_0, B_0, \Delta N)$ is the probability of ΔA being equal to k, given the values of A_0, B_0 and ΔN. The sum may easily be performed numerically. For the moments of the distribution there are, however, also closed-form analytic expressions.

Exact calculation of asymptotic moment values Let $\langle\cdot\rangle_0$ be the average over the initial conditions, $\langle\cdot\rangle_{\Delta A}$ be an average over ΔA, and $\langle\cdot\rangle$ be an average over both quantities. From the properties of the beta-binomial distribution we know that, for a given initial condition, we have

$$\langle \Delta A\rangle_{\Delta A} = \frac{\Delta N A_0}{A_0 + B_0}\,, \quad (4)$$

$$\mathrm{Var}[\Delta A] = \frac{\Delta N A_0 B_0 (A_0 + B_0 + \Delta N)}{(A_0 + B_0)^2 (A_0 + B_0 + 1)}\,. \quad (5)$$

For the mean of $\langle x\rangle$, one obtains

$$\langle x\rangle \overset{(4)}{=} \langle x_0\rangle_0 = \bar{x}_0\,.$$

Hence, the average composition is exactly conserved throughout the time evolution of the populations. In other words, the stochastic process is a martingale.

For the variance we obtain

$$\begin{aligned}
\mathrm{Var}[x] &= \left\langle \left(\frac{A_0 + \Delta A}{A_0 + B_0 + \Delta N} \right)^2 \right\rangle - \langle x_0 \rangle_0^2 && (6) \\
&= \left\langle \frac{A_0^2 + 2A_0 \langle \Delta A \rangle_{\Delta A} + \mathrm{Var}[\Delta A] + \langle \Delta A \rangle_{\Delta A}^2}{(A_0 + B_0 + \Delta N)^2} \right\rangle_0 - \langle x_0 \rangle_0^2 && (7) \\
&\overset{(4)}{=} \left\langle \left(\frac{A_0}{A_0 + B_0} \right)^2 + \frac{\mathrm{Var}[\Delta A]}{(A_0 + B_0 + \Delta N)^2} \right\rangle_0 - \langle x_0 \rangle_0^2 && (8) \\
&\overset{(5)}{=} \mathrm{Var}[x_0] + \left\langle \frac{\Delta N A_0 B_0}{(A_0 + B_0)^2 (A_0 + B_0 + \Delta N)^2 (A_0 + B_0 + 1)} \right\rangle_0 && (9) \\
&= \mathrm{Var}[x_0] + \langle x_0 (1 - x_0) \rangle_0 \left\langle \frac{1}{N_0 + 1} \frac{\Delta N}{N_0 + \Delta N} \right\rangle_0 . && (10)
\end{aligned}$$

For long times (i.e., $\Delta N \gg 1$), $\Delta N + N_0 \simeq \Delta N$ and (10) reduces to

$$\mathrm{Var}[x] \to \mathrm{Var}[x_0] + \left\langle \frac{1}{N_0 + 1} \right\rangle_0 \langle x_0 (1 - x_0) \rangle_0 . \qquad (11)$$

The argument up to here is completely independent of the particular choice of initial conditions. If the initial distribution is known, we may even make the value of the variance more explicit. In particular, consider the distribution we obtain from experiments: in each well, N_0 is Poisson-distributed with mean $\bar{N}_0$. Then one gets

$$\left\langle \frac{1}{N_0 + 1} \right\rangle_0 = \frac{1 - \mathrm{e}^{-\bar{N}_0}}{\bar{N}_0} . \qquad (12)$$

Within each well of (random) size N_0 there is an initial random number A_0 of A-individuals, which follows a Binomial distribution with parameters N_0 and $\bar{x}_0$. For this choice of distribution, it is possible that $N_0 = 0$, which would lead to an undetermined value of $x_0 = A_0/N_0$, and therefore also for the average $\langle x_0 \rangle$. We can solve this problem by redefining x_0:

$$x_0 := \begin{cases} \bar{x}_0 & , N_0 = 0 \\ \frac{A_0}{N_0} & , \text{otherwise} \end{cases} \qquad (13)$$

so that x_0 and its average have definite values, and $\langle x_0 \rangle_0 = \bar{x}_0$. With this we can

compute the second moment of x_0:

$$\langle x_0^2\rangle_0 = \sum_{N_0=1}^{\infty} \mathrm{e}^{-\bar{N}_0} \frac{\bar{N}_0^{N_0}}{N_0!} \left\{ \sum_{A_0=0}^{N_0} \binom{N_0}{A_0} \bar{x}_0^{A_0} (1-\bar{x}_0)^{N_0-A_0} \frac{A_0^2}{N_0^2} \right\} + \bar{x}_0^2 \mathrm{e}^{-\bar{N}_0} \,. \tag{14}$$

The sum inside the braces can be solved using exponential and binomial series and yields

$$\langle x_0^2\rangle_0 = \bar{x}_0^2 + \bar{x}_0(1-\bar{x}_0)\mathrm{e}^{-\bar{N}_0} \sum_{N_0=1}^{\infty} \frac{\bar{N}_0^{N_0}}{N_0! N_0} \,. \tag{15}$$

The remaining series is an exponential integral (Ei), and therefore

$$\mathrm{Var}[x_0] = \bar{x}_0(1-\bar{x}_0)\mathrm{e}^{-\bar{N}_0}\left[\mathrm{Ei}(\bar{N}_0) - \gamma - \ln(\bar{N}_0)\right] =: \bar{x}_0(1-\bar{x}_0)\varphi(\bar{N}_0) \,, \tag{16}$$

where we defined $\varphi(\bar{N}_0) := \mathrm{e}^{-\bar{N}_0}\left[\mathrm{Ei}(\bar{N}_0) - \gamma - \ln(\bar{N}_0)\right]$. Then the variance of x reads

$$\mathrm{Var}[x] = \mathrm{Var}[x_0] + \frac{1-\mathrm{e}^{-\bar{N}_0}}{\bar{N}_0}\langle x_0(1-x_0)\rangle \tag{17}$$

$$= \bar{x}_0(1-\bar{x}_0)\left[\varphi(\bar{N}_0) + \frac{1-\mathrm{e}^{-\bar{N}_0}}{\bar{N}_0}\left(1-\varphi(\bar{N}_0)\right)\right] \,. \tag{18}$$

For large $\bar{N}_0$, through an asymptotic expansion [47]

$$\mathrm{Ei} \simeq \frac{1}{\bar{N}_0}\mathrm{e}^{\bar{N}_0} \sum_{m=0}^{\bar{N}_0-1} m! \bar{N}_0^{-m} - \frac{1}{3}\sqrt{\frac{2\pi}{\bar{N}_0}} \,, \tag{19}$$

$\varphi(\bar{N}_0)$ can be approximated by

$$\varphi(\bar{N}_0) \simeq \frac{1}{\bar{N}_0} \sum_{m=0}^{\bar{N}_0-1} m! \bar{N}_0^{-m} - \mathrm{e}^{-\bar{N}_0}\left[\frac{1}{3}\sqrt{\frac{2\pi}{\bar{N}_0}} - \gamma - \ln(\bar{N}_0)\right] \,. \tag{20}$$

To leading order in $\bar{N}_0$, then, the variance of x becomes

$$\mathrm{Var}[x] = \bar{x}_0(1-\bar{x}_0)\frac{2}{\bar{N}_0} \,,$$

in perfect agreement with our approximate results based on Master equations (Eq. (2) in main text, see also below).

References

47. van Zelm Wadsworth D (1965) Improved asymptotic expansion for the exponential integral with positive argument. Math Comp 19: 327–328.
