# Supplementary material for: Non-Selective Evolution of Growing Populations
Source: PLoS One. 2015 Aug 14;10(8):e0134300. doi: 10.1371/journal.pone.0134300 (PMC4537121; doi:10.1371/journal.pone.0134300)
Supplement: S2 Text — We use the Master equation of the growth process (Eq (1)) to determine the time evolution of variance and skewness of the composition distribution. These values are used in Eq (2) and Fig 2. (PDF) [file pone.0134300.s003.pdf]

S2 Text

Approximate calculations for the time evolution of the distribution moments Using the Master Equation for the number of individuals of each strain (1), we are able to obtain the time evolution of the first three moments of the distribution of x. Equation (1) is sometimes called "Simple Growth Equation" and can be exactly solved (see, for example,[38]) using generating functions like

$$F(a,t) := \sum_{N_A} a^{N_A}\, P(N_A,t). \tag{21}$$

To approximate the time evolution of the first three moments of x, however, we do not need the full solution, but only the first three moments of N_A and N_B. To this end, we insert the Master Equation (Eq. (1) in main text) in the definition of the generating function to get the time derivative for $F(a,t)$:

$$\frac{d}{dt}F(a,t) = \left(-a + a^2\right)\partial_a F(a,t). \tag{22}$$

To obtain the time evolution of the nth moment, we apply the nth derivative with respect to a on both sides of equation (22), and solve for the corresponding moment. For the first three moments, the solution is

$$\langle N_A \rangle = \mathrm{e}^t K_1\,, \tag{23}$$

$$\langle N_A^2 \rangle = \mathrm{e}^t(\mathrm{e}^t - 1)K_1 + \mathrm{e}^{2t}K_2\,, \tag{24}$$

$$\langle N_A^3 \rangle = \mathrm{e}^t\left(-3\mathrm{e}^t + 2\mathrm{e}^{2t} + 1\right)K_1 + 3\mathrm{e}^{2t}\left(\mathrm{e}^t - 1\right)K_2 + \mathrm{e}^{3t}K_3\,. \tag{25}$$

K_1, K_2, K_3 are integration constants, which depend on the initial conditions. We consider the case of Poisson initial conditions. This means that the initial number of A is Poisson-distributed with mean value $\bar{N}_{A,0}$,

$$\langle N_A(t=0)\rangle \overset{!}{=} \bar{N}_{A,0}\,, \tag{26}$$

and, since for the Poisson distribution the variance equals the mean, we get

$$\mathrm{Var}\, N_A(t=0) \overset{!}{=} \bar{N}_{A,0}\,. \tag{27}$$

Employing these conditions in the solutions of the differential equations we found in Eq. (23) and (24), we get

$$\langle N_A\rangle = \mathrm{e}^t \bar{N}_{A,0}\,, \tag{28}$$

$$\mathrm{Var}\, N_A = \mathrm{e}^t(2\mathrm{e}^t - 1)\bar{N}_{A,0}. \tag{29}$$

By the known properties of the Poisson distribution, the skewness of our initial distribution equals to $1/\sqrt{\bar{N}_{A,0}}$. Using Eqs. (25), (28), and the definition of the skewness, we obtain the general time evolution of the skewness

$$v(N_A) = \frac{\bar{N}_{A,0}\left(6e^{2t} - 6e^t + 1\right)e^t}{\left(\bar{N}_{A,0}\left(2e^t - 1\right)e^t\right)^{3/2}}\,. \tag{30}$$

For N_B, the calculations are analogous. Note also that all calculations were exact so far.

With the moments of N_A and N_B we can find the (approximate) time evolution of variance and skewness of $x = N_A/(N_A + N_B)$. For the mean of x we have already seen in the exact calculation (see Eq. (**??**)) that it does not change with time, and hence its time evolution is already known.

To calculate the time evolution of the variance of x, we consider x as a function of N_A and N_B:

$$x(N_A, N_B) = \frac{N_A}{N_A + N_B}. \tag{31}$$

Using the time independence of the mean ($\langle x(N_A, N_B)\rangle = x(\langle N_A\rangle, \langle N_B\rangle)$), a bivariate Taylor expansion around ($\langle N_A\rangle, \langle N_B\rangle$), and the time evolution of the moments, Eqs. (28) and (30), we get for the variance of x:

$$
\begin{aligned}
\operatorname{Var} x &= \langle [x(N_A, N_B) - \langle x(N_A, N_B)\rangle]^2\rangle && (32)\\
&= \left\langle [x(N_A, N_B) - x(\langle N_A\rangle, \langle N_B\rangle)]^2 \right\rangle && (33)\\
&= \langle [x'_{N_A}(\langle N_A\rangle, \langle N_B\rangle)(N_A - \langle N_A\rangle) + && (34)\\
&\quad + x'_{N_B}(\langle N_A\rangle, \langle N_B\rangle)(N_B - \langle N_B\rangle) + \mathcal{O}\left(N_A^{-2}, N_B^{-2}\right)]^2\rangle && (35)\\
&= \frac{\langle N_B\rangle^2}{\langle N\rangle^4}\operatorname{Var} N_A + \frac{\langle N_A\rangle^2}{\langle N\rangle^4}\operatorname{Var} N_B + \mathcal{O}\left(N_A^{-2}, N_B^{-2}\right) && (36)\\
&= \frac{(2-\mathrm{e}^{-t})}{N_0^4} N_{B,0} N_{A,0}\left(N_{A,0} + N_{B,0}\right) + \mathcal{O}\left(N_A^{-2}, N_B^{-2}\right) && (37)\\
&= \frac{2-\mathrm{e}^{-t}}{\bar{N}_0}\bar{x}_0(1-\bar{x}_0) && (38)\\
&\underset{t\to\infty}{\longrightarrow} \frac{2}{\bar{N}_0}\bar{x}_0(1-\bar{x}_0) && (39)
\end{aligned}
$$

From this we obtained Eq. (2) in main text. For infinite times the approximate result for the variance matches the exact one of Eq. (??).

The skewness of the x distribution is calculated analogously:

$$
\begin{aligned}
v(x) &= \left\langle \left(\frac{x(N_A, N_B) - x(\langle N_A\rangle, \langle N_B\rangle)}{\sqrt{\operatorname{Var} x}} \right)^3 \right\rangle && (40)\\
&= \frac{x_0 e^{-2t}\left(12x_0^2 e^{2t} - 12x_0^2 e^t + 2x_0^2\right)}{N_0^2\left(\frac{x_0}{N_0}\left(-2x_0 e^t + x_0 + 2e^t - 1\right)e^{-t}\right)^{1.5}}\\
&\quad + \frac{x_0 e^{-2t}\left(-18x_0 e^{2t} + 18x_0 e^t - 3x_0 + 6e^{2t} - 6e^t + 1\right)}{N_0^2\left(\frac{x_0}{N_0}\left(-2x_0 e^t + x_0 + 2e^t - 1\right)e^{-t}\right)^{1.5}} + \mathcal{O}\left(N_A^{-2}, N_B^{-2}\right)\,. && (41)
\end{aligned}
$$
