# Supplementary figures and images for: Non-Selective Evolution of Growing Populations
Source: PLoS One. 2015 Aug 14;10(8):e0134300. doi: 10.1371/journal.pone.0134300 (PMC4537121; doi:10.1371/journal.pone.0134300)

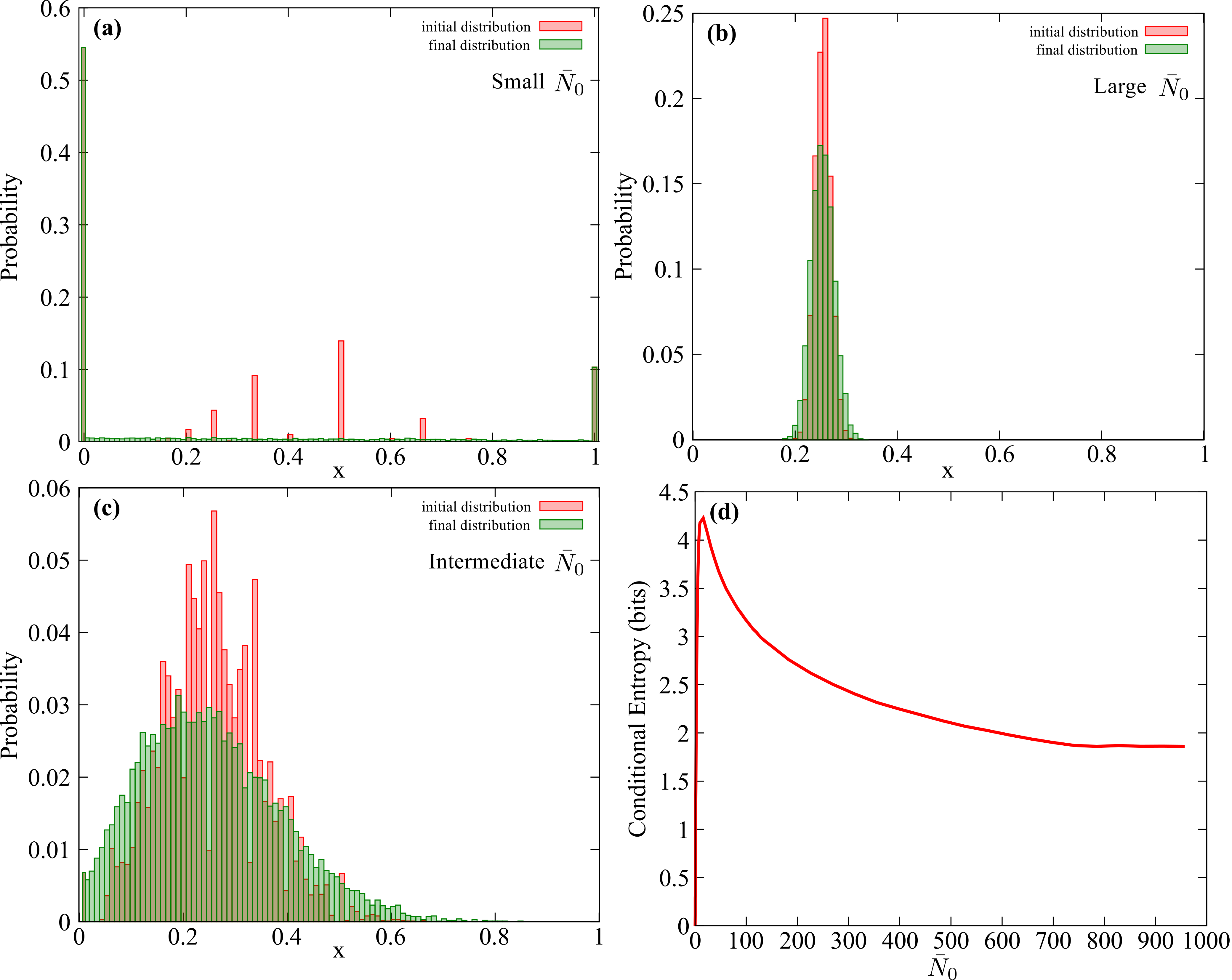

Supplement: S1 Fig — Panels (a),(b),(c): Initial and final distributions of x for three regimes of N‾0. When N‾0 is very small or very large (panels (a) and (b)), the evolutionary fate of the population is largely determined by the initial population sampling. Therefore, the initial distribution (red bars) and the steady-state one (green bars) look qualitatively very similar. For intermediate values of N‾0, however, population growth becomes more important, and the distributions look very different. The amount of composition values the population can access through growth can be quantified looking at the “unpredictability” of the steady-state composition, once the initial one is known: the more unpredictable, the more are made accessible by growth. Mathematically, the measure for this is called conditional entropy: the higher the entropy, the more unpredictable the outcome. Panel (d) shows the conditional entropy as function of N‾0. Indeed, very small or very large initial populations experience little to no additional noise from growth, while in populations with intermediate values of N‾0 (N‾0≃15) growth is a major source of demographic noise. (Parameter values: N‾0=2 (a), N‾0=2000 (b), N‾0=20 (c); x‾0=0.25 in all panels) (TIF) [file pone.0134300.s004.tif]

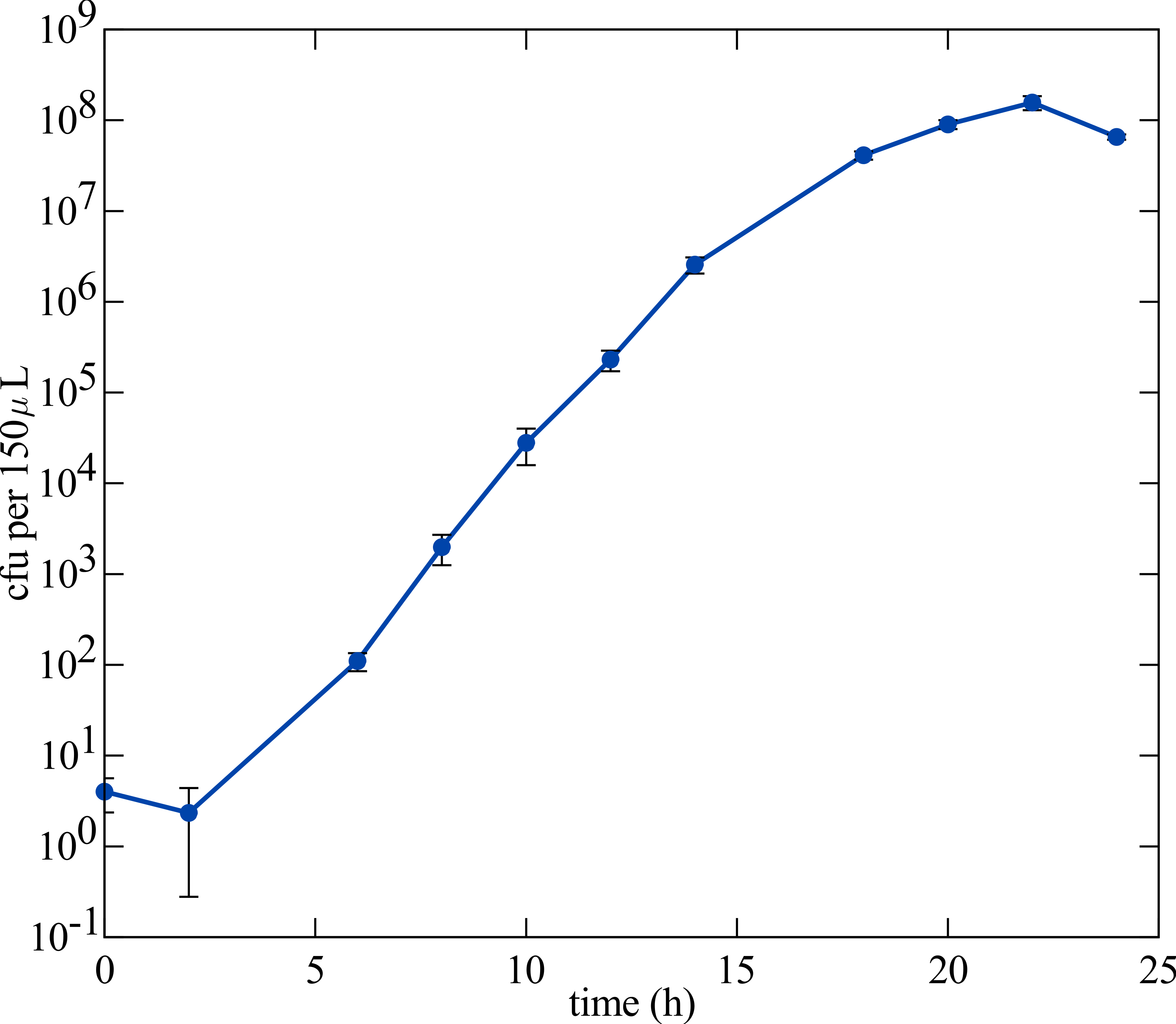

Supplement: S2 Fig — The population consists of pyoverdine producer (P. putida KT2440) and non-producer (P. putida 3E2) under non-selective (iron replete) conditions. Individual precultures of the strains were mixed and diluted in iron replete medium to yield N‾0=4 (in 150 μL), and x‾0=0.5. Cells were grown aerobically at 30°C for 24 hours. The dots represent the mean N(t) of three independent replications, the bars the corresponding standard deviation. After a lag phase of about 2 hours, the cells start to grow exponentially and reach the stationary phase after about 14 h of growth. For the non-selective growth experiments used to test the predictions of the Pólya urn model, cells were grown for 11.5 h to ensure exponential growth conditions. (TIF) [file pone.0134300.s006.tif]

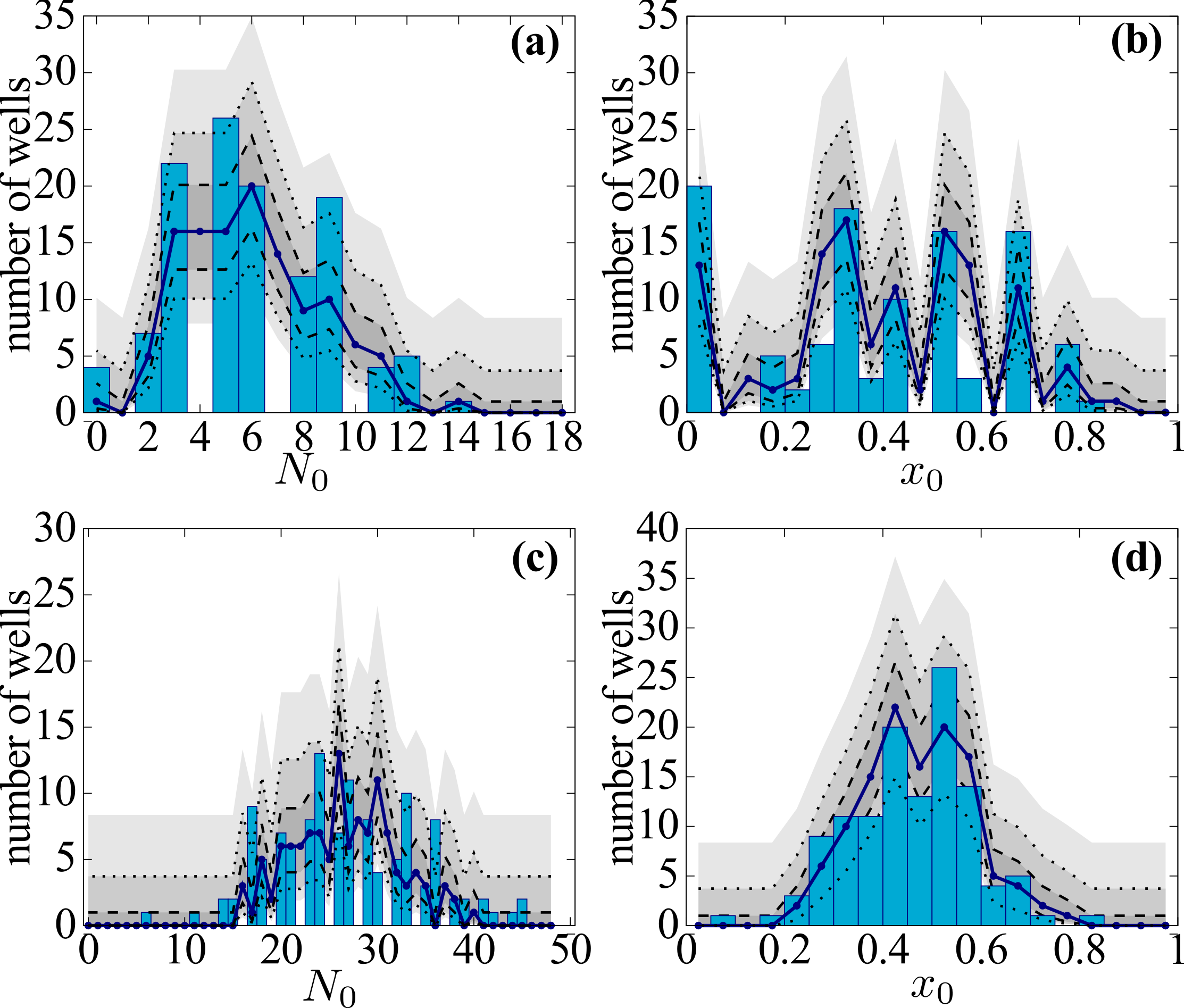

Supplement: S3 Fig — The experimental distributions (bars) are measured from 120-well ensembles, the average N 0 and x 0 from those sets the parameters for the simulated distributions. The theoretical average distribution (solid line) is the average of the same distributions generated for 84 sets of 120 wells. Using that average we calculate three Wilson binomial confidence intervals (gray areas). Experiments and theory agree within statistical error: the distribution of sizes (panels (a) and (c)) follows a Poisson distribution. The raggedness of the distribution of x for at small N‾0 (see panel (b) and Fig 3(b) in main text) is due to a small size effect: since x must be a simple fraction, when N 0 is small only a few values are available (see main text). This effect disappears for average initial sizes N‾0≃10 (see panel (d)). Parameter values: N‾0=5.75, x‾0=0.43 (a) and (b); N‾0=26.49, x‾0=0.45 (c) and (d). (TIF) [file pone.0134300.s007.tif]
