# Supplementary material for: Non-Selective Evolution of Growing Populations
Source: PLoS One. 2015 Aug 14;10(8):e0134300. doi: 10.1371/journal.pone.0134300 (PMC4537121; doi:10.1371/journal.pone.0134300)
Supplement: S3 Text — We use conditional entropy to analyze the impact of growth on the distribution of compositions x. The results are also depicted in S1(d) Fig (PDF) [file pone.0134300.s005.pdf]

S3 Text

Comparison of initial and steady-state distributions of x, and entropy of the steady state distribution conditioned on the initial one. We simulate an ensemble of populations starting from Poisson initial conditions, and track their time evolution until the x distribution freezes. Once it freezes, we can build a joint histogram of initial and final compositions, which approximates the joint distribution $P_{\text{joint}}(x_0, x_f)$. From P_{joint} we can obtain the initial and final distributions as its marginal distributions, integrating over all values of x_f and x_0, respectively. The joint information (Shannon) entropy is defined as [48]

$$H_{\text{joint}}(x_0, x_f) = -\int_0^1 \mathrm{d}x_0 \mathrm{d}x_f P_{\text{joint}}(x_0, x_f) \log(P_{\text{joint}}(x_0, x_f)) \,. \tag{42}$$

The marginal entropies $H(x_0)$ and $H(x_f)$ are defined, analogously, through integrals only of $P(x_0)$ over x_0, and $P(x_f)$ over x_f, respectively. The conditional entropy of the final distribution given the initial is defined as

$$H(x_f|x_0) = H_{\text{joint}}(x_0, x_f) - H(x_0) \,. \tag{43}$$

It measures the amount of information necessary to describe the final distribution, once all information about the distribution of x_0 is known. Therefore, $H(x_f|x_0)$ provides a measure of how entropic (or “noisy”) growth itself is [49]—or, in other words, how many different final compositions are possible given the initial condition. Figure S4**(d)** shows $H(x_f|x_0)$ from repeated simulations, all with the same initial distribution form, the same $\bar{x}_0$, but different $\bar{N}_0$. For very small $\bar{N}_0$ (of the order of one or two individuals) the group formation almost completely determines the fate of populations: most populations start fixated, many with just a single founder individual, and the composition of each well remains the same during growth. The path followed by x in each population during time is a straight line, as the compositions stay constant. Therefore, x for different populations follow in time paths that do not cross or “mix”. Growth produces very little demographic noise, and its conditional entropy tends to zero. For very large $\bar{N}_0$ (of the order of a few hundreds), the group sampling is again central to determine the final distribution. Very large populations, in fact, all start with

similar compositions (according to the Law of Large Numbers), and their compositions are difficult to change, as each individual event has little impact. The composition distribution changes very little before freezing; time evolution paths of different populations “mix” very little. Entropy in this regime saturates for increasing initial sizes, and is rather low. Between the small size regime (where paths do not “mix”) and the large size regime (where size limits “mixing”), we find a window where populations are small enough to significantly change their composition, but also large enough to not start fixated. This is the region where the conditional entropy peaks, and growth is the most important in determining the final distribution.

Intuitively, the difference in variance between initial and final distribution could provide an alternative measure of the noise introduced by growth. However, of all x distributions between 0 and 1 with fixed $\bar{x}_0$, the one with maximal variance is the one for which x is only 0 or 1, i.e., when all populations start off fixated. In this case, the compositions never change during growth and the variance stays constant. Moreover, independently on the choice of initial distribution, the difference between initial and steady-state variance decreases for increasing $\bar{N}_0$ (see Eq.(11)). Therefore, all considerations on noise sources based on variance would indicate that growth matters more when initial populations are smaller, in contrast with our observations.

References

48. Shannon CE (1963) The mathematical theory of communication. MD Comput 14: 306–317.

49. Bialek W (2012) Biophysics: Searching for Principles. Princeton University Press.
