# Supplementary material for: Non-Selective Evolution of Growing Populations
Source: PLoS One. 2015 Aug 14;10(8):e0134300. doi: 10.1371/journal.pone.0134300 (PMC4537121; doi:10.1371/journal.pone.0134300)
Supplement: S1 Table — While experiments for constant-sized populations of Drosophila observe significant fixations within the first tens of generations, we instead observe freezing of the probability distribution for the population composition, without any fixation. (PDF) [file pone.0134300.s008.pdf]

	Drosophila	*P.Putida*
**# of populations**	107	120
**Initial pop. size**	16	~ 10
**Max. # of generations**	19	16
**Pop. size**	Constant	Growing
**Outcome**	Increasing number of fixations	No fixation, freezing
