# Supplementary material for: Non-Selective Evolution of Growing Populations
Source: PLoS One. 2015 Aug 14;10(8):e0134300. doi: 10.1371/journal.pone.0134300 (PMC4537121; doi:10.1371/journal.pone.0134300)
Supplement: S2 Table — Separate cultures of producer (P. putida KT2440) and non-producer (P. putida 3E2) were grown in iron-limiting (no addition of FeCl3) and iron-replete medium (addition of 200 μM FeCl3) at 30°C. The cell density was measured at 600 nm, and specific growth rates were calculated from density values of the exponential phase. The pyoverdine production was determined by fluorescence emission measurements (excitation 400 nm, emission at 460 nm). The pyoverdine production per cell represents the ratio of pyoverdine fluorescence and optical density measured after 24 h of growth. The values in the table are averages over a minimum of five experiments, with the corresponding standard deviation. The fluorescence value for the non-producing mutant in iron-limiting medium is 0 because the culture failed to grow. (PDF) [file pone.0134300.s009.pdf]

	**Specific growth rate (h^{-1})**		**Fluorescence per cell (a.u.)**	
**Iron conc. (μM)**	*KT2440*	*3E2*	*KT2440*	*3E2*
0	0.058±0.006	*no growth*	244.00 ± 21.3	0 ± 0
200	0.152±0.026	0.146±0.017	1.56 ± 0.27	0.93 ± 0.10
